# Leveraging the private sector for child health: a qualitative examination of caregiver and provider perspectives on private sector care for childhood pneumonia in Uttar Pradesh, India

**DOI:** 10.1186/s12913-017-2100-z

**Published:** 2017-02-22

**Authors:** Aurélie Brunie, Rachel Lenzi, Anamika Lahiri, Rasa Izadnegahdar

**Affiliations:** 1 Program Sciences and Technical Support, FHI 360, 1825 Connecticut Ave NW, Washington, DC 20009 USA; 2 Global Health Research, FHI 360, 359 Blackwell St Suite 200, Durham, NC 27701 USA; 3 Independent Researcher, New Delhi, India; 4 Global Health Program, Bill & Melinda Gates Foundation, 500 5th Avenue North, Seattle, WA 98109 USA

**Keywords:** Child health, Pneumonia, India, Private sector, Qualitative research

## Abstract

**Background:**

The private health sector is a primary source of curative care for childhood illnesses in many low- and middle-income countries. Therefore ensuring appropriate private sector care is an important step towards improving outcomes from illnesses like pneumonia, which is the leading infectious cause of childhood mortality worldwide. This study aimed to provide evidence on private sector care for childhood pneumonia in Uttar Pradesh, India, by simultaneously exploring providers’ knowledge and practices and caregivers’ experiences.

**Methods:**

We conducted in-depth interviews with a purposive sample of 36 practitioners and 34 caregivers in two districts. Practitioners included allopathic doctors, AYUSH providers, and drug sellers. Caregivers were mothers of children under the age of five with symptoms consistent with pneumonia who had seen one of those practitioners. Interview transcripts were analyzed thematically.

**Results:**

Caregivers were generally prompt in seeking care outside the home, but many initially favored local informal providers based on access and cost. Drug sellers were not commonly consulted for treatment. Formal providers had imperfect, but reasonable, knowledge of pneumonia and followed appropriate steps for diagnosis, though some gaps were noticed that were primarily related to lack of (or failure to use) diagnostic tools. Most practitioners prescribed antibiotics and supportive symptomatic treatment. Relational and structural factors encouraged overuse of antibiotics and treatment interruption. Caregivers often had a limited understanding of treatment but wanted rapid symptomatic improvements, frequently leading to sequentially consulting multiple providers and interrupting treatment when symptoms improved. Providers were confronted with these expectations and care-seeking patterns.

**Conclusions:**

This study contributes in-depth evidence on private sector care for childhood pneumonia in UP. Achieving appropriate care requires an enriched perspective that simultaneously considers the critical role of provider-caregiver interactions and of the context in which they occur in shaping treatment outcomes.

**Electronic supplementary material:**

The online version of this article (doi:10.1186/s12913-017-2100-z) contains supplementary material, which is available to authorized users.

## Background

In 2013, pneumonia and diarrhea combined accounted for almost a quarter of child deaths worldwide [1]. One of the keys to reducing under-five mortality is addressing barriers to timely, appropriate, and complete treatment, including in the context of private sector health care. Many households in low- and middle-income countries seek curative care for childhood illnesses from formal and informal for-profit providers, particularly in Asia [2–17]. Yet private sector health care provision suffers important shortcomings, including poor adherence to medical standards and incentives for unnecessary care such as the irrational use of antibiotics [18–22]. On the caregiver side, a number of factors such as inadequate knowledge, intra-household dynamics, treatment preferences, or barriers to access in terms of cost and distance may cause delays in seeking care or affect treatment adherence, further contributing to poor treatment outcomes [23–27].

In 2012, India registered the highest burden of child mortality worldwide; pneumonia was the leading cause, claiming over 295,000 lives [28]. Uttar Pradesh is India’s most populous state, and the one with the largest burden of child mortality [29]. The private health workforce comprises formal and informal providers, as well as licensed and unlicensed drug sellers. Formal providers include allopathic doctors mostly located in urban and semi-urban areas, and practitioners of alternative systems of medicine (AYUSH—ayurveda, yoga, unani, siddha, or homeopathy), generally located in rural and small towns [30]. Integrated practice of traditional and allopathic medicine is a long-standing controversy: although AYUSH training covers modern medicine, current legislation in most states does not allow AYUSH providers to practice allopathy. Informal providers are ubiquitous in rural areas. Commonly known as registered medical practitioners or RMPs in reference to a defunct registration system for providers with limited or no formal qualifications, they are designated here as rural health practitioners (RHPs) to avoid confusion regarding their credentials [30, 31]. To our knowledge, little evidence is available on private sector care for pneumonia among children in India. Few studies focus directly on pneumonia and/or take a comprehensive view of the role of the demand and supply sides in shaping care and treatment outcomes. This study aimed to assess formal providers’ and drug sellers’ attitudes and practices related to pneumonia in children and to examine the care-seeking and treatment behaviors of caregivers of children seeking care in the private sector for suspected pneumonia. Informal providers (RHPs) were not included.

## Methods

Uttar Pradesh is organized into 75 districts that are further divided into 311 tehsils (administrative subdivisions). The study was conducted in two tehsils, each being located in a different district. The two districts (Shahjahanpur and Barabanki) were chosen among the 12 intervention districts of another project on private sector treatment of childhood diarrhea, under which listings of all providers and drug sellers had been compiled. The selection of districts and tehsils was purposive, with a view to introduce some geographic and social diversity and some variation in the composition of the private health workforce.

The study included in-depth interviews (IDIs) with practitioners (allopathic, AYUSH, and drug sellers) and with caregivers. A convenience sample of practitioners was selected using listings from the diarrhea project, stratified by tehsil and provider type. Eligible caregivers were mothers aged 18 or older who had accompanied a child under age five with suspected pneumonia to one of the practitioners interviewed. Identification of children with suspected pneumonia was based on a set of symptoms, including reports of 1) a history of cough (longer than 2 days) or difficulty breathing and 2) fast breathing, as per the World Health Organization (WHO) Integrated Management of Childhood Illness clinical algorithm [32]. Throughout the period of fieldwork, treating providers provided research assistants (RAs) with information about caregivers bringing children exhibiting screening symptoms to them who were willing to be contacted for an interview; RAs also asked caregivers about symptoms for confirmation purposes when arranging for the interview. At drug shops, observers directly approached caregivers to determine eligibility, with the drug seller’s permission. In consideration of children being sick, all interviews were scheduled at a later date within a few days of the initial contact with the provider or observer. Following evidence that saturation can occur within 12 interviews and common themes emerge within six [33], we aimed to complete 12 IDIs with each type of practitioner (six per tehsil), 16 IDIs with caregivers for each type of formal provider and up to 10 caregivers for drug sellers (eight and five per tehsil, respectively, with caregivers recruited from at least two different practitioners of each type within each tehsil).

Masters-level RAs hired as consultants conducted interviews in Hindi in December 2013 and January 2014. RAs were organized in two teams, each consisting of three female data collectors and one male supervisor responsible for coordinating arrangements. Three topic guides were developed (caregivers, formal providers, drug sellers—see Additional files 1, 2 and 3); each consisted of a series of broad and follow-up questions for discussion, with RAs instructed to adjust the flow of the conversation to participants’ answers and use prompt to probe for greater depth. Prior to data collection, all RAs participated in an intensive week-long training led by one author (AB), which included a pre-test. Throughout fieldwork, RAs participated in daily debriefs led by a designated member on each team, including one author (AL), and maintained daily email contact with another author (AB) who provided feedback on completed transcripts.

All participants were interviewed individually by a single RA, and in private. Provider IDIs lasted 49 min on average, and caregiver IDIs 35 min. Providers were interviewed at their place of practice and received a token gift (US $8 value) to compensate them for their time. Caregivers were interviewed in their homes, and were not compensated. Verbal consent was sought to avoid potential issues with literacy. IDIs were audio-recorded, and translated and transcribed into English; RAs recorded observations in field notes that were reported in the transcripts. Typed transcripts were uploaded into NVivo 10 for coding and thematic analysis. Each set of transcripts (provider and caregiver) was divided between two analysts who ran periodic checks for intercoder agreement by independently coding the same transcript and comparing results. Codes included a priori codes identified based on informational needs and data-driven codes that emerged from the initial reading of transcripts. We developed detailed memos describing the dimensions of each main code, then used Excel matrices to examine the prevalence of key themes and the relationships between them and/or with participants’ characteristics. The Institutional Review Board at the Centre for Media Studies in New Delhi and FHI 360’s Protection of Human Subjects Committee in the USA approved this study.

## Results

Thirty-six IDIs were completed with providers, and 34 with caregivers (Table 1). During the recruitment process, four providers refused to participate in the study and two who had agreed to an appointment could not be reached at the scheduled time. In one tehsil, RAs were only able to identify and recruit four caregivers from allopathic providers (instead of eight). Though RAs attempted to confirm symptoms before the interviews, three IDIs with caregivers were excluded from analysis because narratives were not consistent with acute respiratory infection (ARI) episodes.

**Table 1:** Number of IDIs conducted, by participant group

	Shahjahanpur	Barabanki	Total
Providers			
Allopathic	6	6	12
AYUSH	6	6	12
Drug sellers	6	6	12
*Total*	*18*	*18*	*36*
Caregivers recruited from:			
Allopathic	8	4	12
AYUSH	8	8	16
Drug sellers	3	3	6
*Total*	*19*	*15*	*34*

Participant characteristics are shown in Tables 2 and 3. Almost all practitioners were men, and the majority of formal providers dispensed drugs. Caregivers had an average of 2.4 children; most mothers were illiterate. The mean age of children with suspected pneumonia in our sample was 18.7 months; about two-thirds were boys.

**Table 2:** Provider characteristics (*N* = 36)

	Allopathic	AYUSH	Drug sellers
Gender			
Male	11	11	12
Female	1	1	0
Mean age (years)	60	46	38
Mean number of years of experience	29	20	13
Designation			
	Generalist 9	Ayurvedic 5	Licensed 4
	Pediatrician 3	Homeopath 4	Unlicensed 8
		Unani 3	
Dispensing			
Yes	7	9	12
No	5	3	0

**Table 3:** Caregiver characteristics (*N* = 31)^a^

	Total
Mean age of mother (years)	25.4
Highest level of education achieved	
None, illiterate	17
None, literate	0
Standard 1-5	5
Standard 6 or higher	9
Religion	
Hindu	19
Muslim	10
Other	1
Caste/tribe	
Scheduled caste	6
Scheduled tribe	2
Other backward class	11
None	2
Not disclosed	2
Mean number of children in care	2.4
Gender of child with suspected pneumonia	
Male	21
Female	10
Mean age of child with suspected pneumonia (months)	18.7

^a^This table does not include the three IDIs that were excluded from analysis

We collated codes from the analysis of caregiver and practitioner transcripts into two broader emerging themes to explain the data: 1) care-seeking pathways to private providers, and 2) private sector case management strategies.

### Care-seeking pathways to private providers

#### Decision to seek care outside the home

When asked how they noticed that their child was sick, most caregivers mentioned a cough and/or difficult breathing. Among those, over a third also reported fever and a quarter reported fast breathing or intercostal retractions. In the majority of cases, symptoms were readily attributed to *sardi* (cold) or, in Barabanki, *jakda* (congestion), though those two conditions were not strictly defined or necessarily seen as mutually exclusive. More broadly, several mothers understood pneumonia as a more serious cold. Illustrating the ambiguity in illness typology and recognition of severity, the 27-year old mother of a 3-month old boy said:

*“[Sardi, jakda, pneumonia] It’s all the same thing…If it gets bad, then one says that sardi-pneumonia has happened, take him to the doctor quickly. He’s caught jakda, he’s caught pneumonia. If it’s slight, one says that it’s sardi, put something warm on him, like oil…If it’s slight, they play, they’re active and roam around. If it’s bad, they stop playing and walking around, [they] stay inside.” (2143)*

Mothers typically reported seeking care outside the home within 1 to 4 days of first noticing symptoms. One third of caregivers mentioned a single specific symptom as the trigger for this decision and two thirds a suite of symptoms. A number of mothers referred to the failure of home remedies to improve the child’s condition as a trigger. Worsening in the ability to breathe properly was the most often reported sign interpreted as indicative of severity or disease progression throughout caregiver interviews; however, where mothers elaborated, descriptions of the symptoms underlying this appreciation were heterogeneous, ranging from a congested nose through chest sounds to intercostal retractions.

A third of formal providers, mostly AYUSH, said that caregivers, particularly in rural areas, delayed seeking medical help, mostly because they failed to understand that the condition could progress and attempted to take care of it at home first, minimizing spending.

#### Sequence of care

Caregivers indicated consulting “village” doctors, including RHPs and possibly other doctors practicing locally, and AYUSH practitioners much more often than they did allopathic providers. Caregivers who were illiterate disproportionately reported selecting village doctors. Two of the six caregivers recruited from drug sellers used the drug seller as first point of contact.

Most mothers did not know or provide details on providers’ credentials or on the system of medicine the providers practiced; they typically referred to all providers, including drug sellers, as “doctors.” At the same time, caregivers were not oblivious to the fact that there were differences between providers. For instance, several caregivers who initially consulted a village doctor or an AYUSH practitioner made a conscious decision to experiment with those providers for initial care, as illustrated here by the mother of a 4-year old boy who consulted a unani doctor:

*“We show our child to Dr. D [unani doctor] only, first of all. When it is too much, then only we go that nursing home…We think that the child maybe [will] get relief from here so that we do not need to go too far…There is a lot of difference [between the unani doctor and the nursing home]…We feel that D is cheaper…We also think that he is a person of our own home; he will give good medicine.” (1231)*

Convenience, prior experience with the provider, and cost were the most commonly mentioned drivers of initial provider choice. Over half of caregivers reported consulting multiple providers over the course of the ARI episode, typically two and sometimes three by the time of the interviews; this was mostly due to the perception that their child was failing to “get relief” after seeing the initial provider. A few switched providers when there was no improvement within a day or so, but more waited between 3 and 5 days. Retracing care pathways from the caregiver interview narratives shows that caregivers mostly switched “upwards” to providers with higher levels of qualification. For example, almost all caregivers whose child initially consulted a village doctor went on to an AYUSH practitioner or allopathic doctor.

In most cases, both mother and father took the child to the provider together. Many caregiver interviews portrayed men as financial gatekeepers who arranged for money and were responsible for financial transactions, including payments to providers and medicine procurement; a few mothers also indicated that they never visited a provider unescorted. Whether initially or at a later point, over a third of caregivers indicated that they had received advice from relatives (beyond the husband) in choosing providers, often from a brother/brother-in-law or mother/mother-in-law.

All allopathic providers and three-quarters of AYUSH doctors said that caregivers often came to them after first consulting a village-level provider; of those, a third specifically mentioned RHPs. Many providers identified access and cost as the main determinants of initial care-seeking decisions, though several providers, mostly allopathic doctors, also cited poor health education, which resulted in a failure to understand both the seriousness of the child’s condition and the lack of qualifications of RHPs. Several providers, mostly allopathic ones, said that the escalation of symptoms was a trigger for seeking additional care, with a few noting that village doctors may refuse to continue treatment and/or may refer children to them.

### Private sector case management strategies

#### Provider knowledge base and disease management practices

Table 4 summarizes key dimensions of provider knowledge regarding symptoms, markers of severity, and etiology. One quarter of drug sellers did not know any signs of pneumonia. According to provider interviews, in most cases the reported diagnostic approach involved a brief illness history, a visual physical examination, and auscultation with a stethoscope. Though many providers, particularly allopathic doctors, reported checking vital signs like temperature, breathing rate, and pulse, this was mostly done by hand and appeared to involve judgment calls more than actual measurement. AYUSH doctors often described crepitations and chest in-drawing as the basis for differential diagnosis, while allopathic providers were more likely to say they relied on the overall clinical picture.

**Table 4:** Key aspects of provider knowledge^a^

	Allopathic (*N* = 12)	AYUSH (*N* = 12)	Drug sellers (*N* = 12)
Symptoms of pneumonia			
Difficult breathing	12	12	8
Rapid breathing	11	8	3
Fever	11	6	7
Cough	6	8	6
Markers of severity			
Difficult breathing	7	8	Topic
Fast breathing	8	4	not
At least one danger sign^b^	11	8	included
Etiology			
Viral	10	5	Topic not
Bacterial	7	2	included

^a^The table shows the number of provider interviews in which a particular response was given

^b^Danger signs include unconsciousness/lethargy, lack of appetite/difficulty drinking milk, and cyanosis

Treatment practices are presented in Table 5. Formal providers often mentioned multiple antibiotics, including members from different classes, but mostly prescribed a single pharmaceutical for mild cases. Several providers reported adjusting treatment based on response. Structural and interpersonal constraints to prescribing behavior were noted in several interviews, including lack of equipment to ascertain diagnosis, prior treatment by village doctors, costs, and pressure to relieve symptoms. An allopathic provider said:

*“In a private set-up, you can’t…just admit the patient…without any treatment, just investigations are going on. He’ll say I’m wasting my money. And if by the way you are wrong at a diagnosis and you end up with complications, he’ll just think that you did not give any treatment, that’s why my child is [in a] serious [condition]. You have to give a prophylactic antibiotic. In a private practice, what I’m doing is, we are giving oxygen, bronchodilator, and an antibiotic.” (213)*

**Table 5:** Treatment practices among formal providers, as reported by providers

	Allopathic (*N* = 12)	AYUSH (*N* = 12)
Use of antibiotics	12	7
Antibiotics commonly reported as prescribed^a^		
3rd generation cephalosporin	6	6
Amoxicillin	4	2
Amoxicillin-clavulanic acid	3	3
Azithromycin	3	3
Amikacin	3	3
Use of supportive treatment^b^	10	8
Integrated practice of traditional and allopathic medicine	N/A	
Antibiotics only		5
Antibiotics and Indian medicine		2
Indian medicine only^c^		4

^a^Only the most frequently mentioned antibiotics are shown. Providers may mention antibiotics from more than one class

^b^Examples of supportive treatment included antipyretic, bronchodilators, and steroids

^c^One provider indicated prescribing only supportive treatment

Three quarters of formal providers said that they referred cases that they deemed too severe upon initial presentation, and half that they referred mild cases when the child did not respond to the treatment. Over half of formal providers reported referring cases when they did not have adequate equipment or facilities to handle them, with lack of pediatric expertise as another common reason. Though most providers listed multiple symptoms triggering referral, all but one said that difficulty breathing was a deciding factor. Two-thirds said that treatment of severe pneumonia required nebulization and/or oxygen for respiratory support (although nebulization was not limited to severe cases), with allopathic providers mentioning both and AYUSH doctors primarily talking about nebulization. Oxygen was rarely available, but a number of providers said they had nebulizers. Several AYUSH providers also reported referring cases because they did not prescribe allopathic medicine or particular antibiotics or forms of drugs (like injectables).

Most drug sellers saw children as high-risk cases. Half of drug sellers reported prescribing for children with respiratory illnesses, while the other half said that they did not. All but one of the prescribing drug sellers said they only handled minor cases that they felt were within their capacity. Several drug sellers advised caregivers on when it was necessary to see a doctor, whom to consult, and/or helped making contact with providers, negotiate fees, or relay advice. When filling prescriptions, half of drug sellers said that they sometimes gave substitutes: two consulted the prescribing provider; three identified appropriate substitutes based on composition when they were out of a particular medicine (though one sometimes changed the dose); and one substituted cheaper drugs when patients were poor.

#### Adherence to prescribed therapy

Most caregivers recruited from formal providers reported multiple visits to the formal provider by the time of the interviews, often to receive injections or nebulization or to procure more drugs. Many described the treatment received as “medicines” or “drugs” but did not supply drug names, though most did differentiate by delivery form when prompted for more details. Some caregivers indicated what symptoms a medicine was for, but most did not describe a difference in the purpose of the medicines. Only one mother mentioned the term “antibiotics.”

Over half of caregivers who consulted one of the formal providers reported discontinuing visits and/or medicines once they felt their child’s condition was improving. Almost all attributed improvements to the effect of “good” medicine, but interpretations of an improved condition were split between positive behaviors like playing, improved appetite or laughing and the lessening, though not necessarily the absence, of negative symptoms associated with sickness.

In some cases, mothers reported being told by the provider to only return if the child got unwell again; but in others, caregivers themselves decided to interrupt the treatment. Several caregivers felt that procuring more drugs was no longer necessary, particularly in light of financial and logistical constraints; others did not finish all the medicine that they had purchased. Five caregivers, however, had resumed visits to the provider after a few days because symptoms had returned or increased again.

Two-thirds of formal providers described follow-up strategies requiring multiple visits to monitor the child’s condition (primarily allopathic doctors) and/or dispense more drugs (mostly AYUSH doctors). Most noted that caregivers did not systematically come back. According to providers, the main reasons for this behavior were switching to other providers if expectations for fast relief were not met, financial constraints, and perceived resolution of illness. Allopathic providers charged a consultation fee that typically covered any visit made within a period of 5 days, while AYUSH providers rarely charged a consultation fee but only gave a few days’ worth of medicine at each visit. An allopathic provider said:

*“Ten to 20% of people only are able to [afford] the full course of treatment. So as soon as they start feeling comfortable, they stop coming…they don’t know whether they’re cured or relieved. They don’t know the difference…the capacity to pay is very low. So for that we’ve planned something. For Rs. 50, for 5 days, they can come and avail of free consultation…they come on one day and pay, then all they need to do is buy medicines on the other days. Because of this, follow-up is comparatively better.” (214)*

## Discussion

This study aimed to identify areas of improvement for ensuring appropriate care for pneumonia among children through the private sector in Uttar Pradesh. On the supply side, formal providers displayed imperfect, but reasonable, knowledge of the signs of pneumonia, including danger signs. The diagnostic approach was also generally appropriate and consistent with WHO recommendations. The main gaps were in cursory examination practices that often did not involve rigorous measurement of vital signs, particularly among AYUSH providers, as well as lack of access to or use of more advanced tools to ascertain the etiology of pneumonia. Drug sellers had limited familiarity with pneumonia, but typically did not manage anything beyond minor illnesses.

On the demand side, care-seeking delays are well-documented in the literature [23, 34–37]; in South Asia research has pointed to gaps in recognition and interpretation of illness [38, 39], structural barriers such as cost of care and distance to facilities [38, 40–42], social negotiations around seeking care outside of the home [41], and gender biases favoring male children [42–44] as factors contributing to delays. In our sample, despite a tendency to try home remedies first, caregivers were generally prompt in seeking outside care, which echoes findings from a study on care-seeking for young infants in a New Delhi urban slum [24].

Some of the most critical gaps identified in achieving appropriate care can be traced to the interactions between caregivers and providers. Findings from both provider and caregiver interviews highlight the complexity and interrelatedness of disease management decisions and practices on either side, and the importance of the wider geographical and economic context. First, caregivers have access to a number of private providers who offer a wide and diverse range of treatment options but are generally affordable and conveniently located. Second, important gaps in caregiver knowledge remained regarding recognition of pneumonia and markers of severity. Third, caregivers do not have clear treatment expectations. In particular, they tend to expect rapid improvements in their child’s condition while having a limited understanding of the treatments prescribed. As a result, treatment decisions were essentially a dynamic process characterized by experimentation with multiple providers and interrupted treatments, and constrained by access and poverty. Other studies similarly report on caregivers’ reliance on a “middle layer” of providers as the first line of care, and on provider shopping and switching when there is no immediate relief [23, 24].

The combination of both caregivers’ and providers’ experiences and the inclusion of several cadres in the sampling strategy offer an enriched perspective. Moreover, the commonalities and complementarities in the theme structure across providers and caregivers provides confidence in the results. There are, however, some limitations to this study. We relied on provider feedback and quick verbal reports on child symptoms to recruit eligible caregivers. While this approach is more standardized than relying on a diagnosis of pneumonia by providers, our sample may have included caregivers of children with other ARIs besides pneumonia. Findings on management of severe pneumonia must be interpreted cautiously due to differences in what providers may consider to be severe. While neither providers nor caregivers discussed gender biases or preferences as factors affecting care-seeking patterns, it is worth noting that 2/3 of the caregivers in our sample were seeking care for a male child, which may indicate intrinsic biases. In light of previous research in India pointing to differentials in health seeking and health outcomes—including mortality—favoring male children [43–46], this topic bears further research and monitoring to determine the extent to which it may influence timing of care seeking and choice of provider. Additionally, we focused on caregivers who made contact with qualified private providers and drug sellers. Thus important insights related to broader recognition of pneumonia and care-seeking patterns are likely not to be fully captured. Information on village doctors is limited to the perspectives of competitors or of caregivers who either chose not to use their services or subsequently required additional assistance. Given their role at the front line, more research is needed for a better understanding of the role, competence, and practices of this cadre.

## Conclusion

This study shows that understanding interactions between caregivers and providers and the context in which they occur is important in developing models of appropriate care. Future research and programs should simultaneously address both the demand and the supply side for better outcomes. In UP, achieving appropriate care for childhood pneumonia through the private sector requires a better alignment of business incentives with recommended standards of care for providers. This should be supplemented by health education and behavior change interventions to enable caregivers to make better decisions for optimal timing and choice of source of care and improve adherence to treatment: the focus should include early recognition of markers of severity, understanding that bacterial pneumonia is time-dependent and can rapidly become life-threatening, and appropriate treatment expectations.

## Additional files

Additional file 1: IDI guide_caregivers_FHI 360: Topic guide for IDIs with caregivers. (PDF 298 kb)
Additional file 2: IDI guide_private providers_FHI 360: Topic guide for IDI with formal providers. (PDF 299 kb)
Additional file 3: IDI guide_drug sellers_FHI 360: Topic guide for IDI with drug sellers. (PDF 291 kb)

## Abbreviations

ARI: Acute respiratory infection
AYUSH: Ayurveda, yoga, unani, siddha, or homeopathy
DAZT: Diarrhea Alleviation through ORS and Zinc Therapy
IDI: In-depth interview
RA: Research assistant
RHP: Rural health practitioner
RMP: Rural medical practitioner
WHO: World Health Organization
