# Supplementary material for: Leveraging the private sector for child health: a qualitative examination of caregiver and provider perspectives on private sector care for childhood pneumonia in Uttar Pradesh, India
Source: BMC Health Serv Res. 2017 Feb 22;17:159. doi: 10.1186/s12913-017-2100-z (PMC5322628; doi:10.1186/s12913-017-2100-z)
Supplement: Additional file 1: — IDI guide_caregivers_FHI 360: Topic guide for IDIs with caregivers. (PDF 298 kb) [file 12913_2017_2100_MOESM1_ESM.pdf]

Assessment of child pneumonia diagnosis and treatment by private providers in Uttar Pradesh, India

In-Depth Interview with caregivers

Introduction

Thank you for agreeing to participate in this study. As I explained a few minutes ago, this research is to better understand why people go to see private providers when their children have respiratory problems, and how they take care of their children when they are sick. The information we learn will help find ways to better help sick children in Uttar Pradesh. We would like to talk with you today because you took your child to ______ recently for a respiratory problem.

General background

Before we get started, I would like to talk about yourself.

- Could you tell me a bit about yourself and about your life?
    a. *Socio-demographic characteristics*
    b. How many children do you care for?
    c. What is your role in taking care of children when they are sick in this household?
        i. Who takes the decision to get outside medical help for the child?
        ii. Who takes the child when they need to get outside medical help?

Patterns of careseeking

Now I would like to ask you some questions about when the child got sick with a respiratory problem.

- What did you think the problem was when you first realized that something was wrong with the child?
- What traditional medicines or home remedies, if any, did you try to use to make the child feel better?
    a. Probe for specific symptoms the remedies were used to treat
- At what point did you decide that the child needed care outside of the home? What did you first notice about the child that made you realize this? Explain.
    a. *Probe for specific symptom and duration*
- Before you went to ____, did you seek help for the child anywhere else?
  If yes:
    a. Where did you go?
        i. *Try to establish whether the person is traditional healer, ASHA, drug seller, private provider (clarify allopathic, unani, homeopath, ayurvedic), or public sector provider (clarify level – health facility or hospital).*
    b. Why did you decide to go there (instead of going directly to _____)?
    c. What advice or services did you receive?
        i. What were you told the problem was with the child?
        ii. What were you told to do to make the child feel better?
        iii. Did this advice influence what you did to take care of the child? Why or why not?
    d. After this, did you take the child to see anybody else before you went to ___?
        i. *If yes, repeat questions a to c*
- Why did you decide to seek help from _______?
    a. Had there been any changes in how the child felt? Explain. *Probe for specific symptoms and duration that caused action.*
    b. Who, if anyone, recommended that you go to____? Explain.
    c. What made you decide to go to ____ and not somewhere else?
    d. Did you bring the child along when you went to ____? Why or why not?

Quality of care

- What did ___ tell you the problem was with the child? How do you call this illness in the community?
    a. What kind of questions did ___ ask you about the child?
    b. What exams or tools did ___ use?
    c. How well did you understand what ____ told you was wrong with the child?
    d. How severely ill did ___ tell you the child was? Explain.
- What drugs, if any, did ___ recommend or administer for the child?
  If drugs were prescribed or administered
    a. *Try to find out name or description of drugs/combination of drugs, dose, and duration. If possible, ask to see drug at the end of the interview and note name, composition, and dose*
    b. How long did ___ tell you to give the drug(s) to the child?
    c. Did ___ give you the drugs, or did you go somewhere else to get the drugs?
       If got drugs elsewhere:
        i. Where did you get the drug(s)? Why did you get the drug(s) there?
    b. Did you get (all) the drug(s) that ____ recommended?
       If not:
        i. Why not?
        ii. Which drug(s) did you get? How did you decide?
- Did ___ recommend or administer other things to the child?
- What other things did ___ tell you to do at home to make the child feel better?
    a. What signs, if any, did ___ ask you to look for to know if the child was getting better?
    b. Did ___ tell you that you should come back with the child for another visit?
        i. When or under what circumstances did ___ tell you that you should come back?
- What, if any, questions did you have? Did you ask those questions? How well were those questions answered?
- What cost did you incur to go to ___ and come back home? How much did you pay for the visit?
    a. *Travel cost, consultation fee, drug cost*
    b. How did you pay?
        i. *Timing (in full or delayed, and when), forms of payment*
    c. *If drugs purchased elsewhere, repeat questions for drugs to establish amount and mode of payment*

Adherence to advice and treatment

- How have you been taking care of the child since you went to ___?
    a. What drug(s), if any, did you give to the child? For how long?
        i. *If drugs had been prescribed*: *compare to what had been recommended and clarify potential discrepancies if drug(s) taken longer/shorter*
        ii. *If no drug(s) had been prescribed*:
            - What made you decide to give this drug to the child?
    b. What other things have you been doing to take care of your child? *Probe for continuation of home remedies, or new home remedies.*
    c. What are some of the challenges that you have encountered in taking care of the child like you had been told to? Explain.
        i. Was there anything that you were told to do but you didn't actually do? Tell me more about what happened.
- Did you go back to ____?
    a. *If yes:* What were the circumstances. Tell me what happened.
    b. *If no*: Why not?
- Did you take the child anywhere else for additional help?
    a. Why did you feel that the child needed additional help?
    b. Why did you decide to go there (instead of going back to ____)?

    c. What kind of help did you receive?

Final words

- Overall, what are the main child health problems in this area during the cold season?
    a. Do young children die in this area? What do they die from?
    b. In your opinion, how dangerous are respiratory illnesses compared to other child illnesses?
- Whose advice do you listen to in terms of health matters for children?
    a. Whose opinions were most important to you in deciding how to take care of the child when he/she got sick with this respiratory problem?
- What information could help you better take care of the child if he/she gets sick with a respiratory problem again in the future?
    a. How would you like to receive this information?
- What is your opinion about the quality of services for child respiratory illnesses in this area?
    a. What could be done to improve the quality of those services?
- Overall, what are the main challenges that your family faces in accessing treatment for child respiratory illnesses?
- Do you have any final questions or recommendations to make on what can be done to improve the health of children in this area?
