# Supplementary material for: Leveraging the private sector for child health: a qualitative examination of caregiver and provider perspectives on private sector care for childhood pneumonia in Uttar Pradesh, India
Source: BMC Health Serv Res. 2017 Feb 22;17:159. doi: 10.1186/s12913-017-2100-z (PMC5322628; doi:10.1186/s12913-017-2100-z)
Supplement: Additional file 2: — IDI guide_private providers_FHI 360: Topic guide for IDI with formal providers. (PDF 299 kb) [file 12913_2017_2100_MOESM2_ESM.pdf]

Assessment of child pneumonia diagnosis and treatment by private providers in Uttar Pradesh, India

In-Depth Interview with private providers

Introduction

Thank you for agreeing to participate in this study. As I explained a few minutes ago, this research is to better understand why people go to see drug sellers and private providers when their children have respiratory problems, how private providers diagnose and treat these problems, and how caregivers then apply the advice they receive. The information we learn will help find ways to better help sick children in Uttar Pradesh. We would like to talk with you today about your experiences with children with respiratory illnesses.

General background

Before we get started, I would like to talk about yourself.

- Could you tell me a bit about yourself and about your experience as a provider?
    a. *Socio-demographic characteristics*
    b. How long have you been working in this position?
    c. What are the different places where you currently practice? *Probe for public vs. private*
    d. What services do you currently provide?
- How does the community learn about you?
- How often do people come to see you about illnesses for children under the age of five?
    a. What are the other places where they go to access treatment for child illnesses?
    b. To what extent do they sometimes go see someone else for child illnesses before they come see you?
        i. For what types of child illnesses do they go to see someone else before they come to you?
        ii. Who do they go to?
        iii. What do you think are the reasons why they do not come to see you directly?

Respiratory illnesses and diagnosis

Thank you. Now I would like to talk specifically about your experience with children with respiratory illnesses.

- What different respiratory illnesses do you see among children under the age of five? At what time of the year do you see the most cases?
- In your experience, how advanced would you say the respiratory illness typically is when caregivers come to consult you?
    a. What are the main reasons why you think caregivers may delay consulting you about their child? Explain.
- What questions do you typically ask caregivers to help you determine what respiratory illness the child has?
- What are the different physical signs that you examine the child for?
    a. What job aides or diagnostic tools, if any, do you use to screen for those signs?
        i. *Probe for tools they may use for each of the different signs they identified*

Pneumonia understanding and perceptions

Thank you. Now I would like to talk specifically about pneumonia.

- How often do you see children with pneumonia as opposed to other respiratory illnesses? At what time of the year do you see most cases?
    a. How do you know when a child has pneumonia and not some other respiratory illness?
    b. What causes pneumonia? Anything else?
        a. How does a child develop pneumonia? *Explore progression/stages if infection mentioned.*
    c. Are there different types of pneumonia?
        i. *If yes:* What are the differences between them?
- How do you determine how severely ill a child with pneumonia is?
    a. What are the signs that indicate that a case is severe to you?

   b. What job aides or diagnostic tools do you use to make this determination?

Pneumonia treatment

- Do you recommend different treatments for different causes of pneumonia?
    a. <u>*If yes*</u>*:* What are the main differences between the treatments that you recommend?
        i. *Probe for drugs vs. no drugs, alone or in combination, duration, dose*
        ii. In what ways are the drugs or combinations of drugs different?
- In what ways is the treatment of severe pneumonia different from the one you recommend for other, less severe cases?
    a. *Probe for drugs vs. no drugs, alone or in combination, change in drugs or combinations of drugs, change in duration, change in dose, oxygen. Probe for name of drug and salt. If possible, ask to see drug or sample at the end of the interview and note name, composition, and dose*
    b. How long does the treatment for severe pneumonia need to be continued?
        i. *Probe for duration for all different aspects of treatment mentioned (e.g. drugs/combination of drugs, oxygen)*
    c. What do you think are the chances that a child with severe pneumonia will make a full recovery? Tell me why you feel this way.
    d. Have you ever seen a child with pneumonia that was so severe that you did not feel that you could help that child on your own, without additional support?
        i. What are the circumstances in which you seek support for a child with severe pneumonia? *(Additional assessments of respiratory rate, chest in-drawing, oxygen levels; provision of oxygen etc.)*
        ii. Where do you seek support? *Establish sources of support, including referrals and clarify type (public vs. private and level – health facility, hospital; or private provider type – allopathic, ISMP)*
            - *For referrals:* How do you decide where to refer people? *Cost, severity etc.*
- Can you tell me about any advice you give caregivers about how to take care of a child with pneumonia at home?
    a. What do you tell caregivers to do at home?
    b. What signs, if any, do you tell them to look out for?
    c. In what ways, if at all, is the advice you give to caregivers different for severe cases compared to milder cases of child pneumonia?
    d. Under what circumstances do you tell caregivers that they should come back to see you?
        a. To what extent do they actually come back when you tell them to?
        b. What do you think are some of the reasons why they may not come back?

Drug dispensing

- Do you dispense drugs?

<u>*If yes*</u>*:*

- Where do you get the drugs for children with pneumonia that you dispense?
    a. Do you use multiple suppliers? Who are they? *Establish whether single vs. multiple suppliers, and supplier type (wholesaler, retailer, visits by sales representatives or drug companies)*
    b. What do you like about your current supplier(s)? What do you not like?
- How do you decide what drugs to buy? What are the different factors that you consider when making this decision?
- Have there been times when you wanted to dispense some drugs to someone caring for a child with pneumonia but you were out of stock? When was the last time this happened?
    a. What were the circumstances?
    b. What did you do?
        i. <u>*If referral*</u>*, establish point of referral – drug sellers, public facility, other private provider*

Amoxicillin

- Have you ever prescribed amoxicillin for any adult or child illness?

If yes:

  a. What illnesses did you prescribe it for?
  b. Under what conditions do you prescribe amoxicillin as first line of treatment for child illnesses?
     i. *If prescribed for children:* What delivery form(s) of amoxicillin do you typically prescribe for children (syrup, dry powder for reconstitution with water to form a suspension, dispersible tablet)? Tell me why you prescribe this form.
        - *Cost, convenience, acceptability*
- In general, what do you think is the most suitable delivery form for drugs for children (e.g. syrup, drops, powder, tablets, dispersible tablets)?
  a. What do you think are the advantages of dispersible tablets over other delivery forms?
  b. What are the disadvantages?

Cost structure

- What is the range of your fees for the treatment of mild pneumonia in a child?
  a. *Clarify fee structure for dispensers, clarifying consultation vs. drugs*
  b. Are your fees different for severe cases of child pneumonia? Explain.
  c. To what extent are your clients able to pay you? Explain.
  d. What forms of payment do you accept?

Final words

- Where do you get your information about child illnesses?
  a. What are your sources of information about treatment options for respiratory illnesses specifically?
  b. What would be the best way for you to receive updates on treatment options given your current busy schedule?
- Overall, what do you find most challenging about knowing the best way to help children with respiratory illnesses?
  a. What additional information or tools would help you in the diagnosis of respiratory illnesses among children?
  b. What additional information or tools would help you treat respiratory illnesses among children?
- Do you have any final questions or recommendations to make to improve the management of child respiratory illnesses in this area?
