# Supplementary material for: Leveraging the private sector for child health: a qualitative examination of caregiver and provider perspectives on private sector care for childhood pneumonia in Uttar Pradesh, India
Source: BMC Health Serv Res. 2017 Feb 22;17:159. doi: 10.1186/s12913-017-2100-z (PMC5322628; doi:10.1186/s12913-017-2100-z)
Supplement: Additional file 3: — IDI guide_drug sellers_FHI 360: Topic guide for IDI with drug sellers. (PDF 291 kb) [file 12913_2017_2100_MOESM3_ESM.pdf]

# Assessment of child pneumonia diagnosis and treatment by private providers in Uttar Pradesh, India

In-Depth Interview with drug sellers

**Introduction**

Thank you for agreeing to participate in this study. As I explained a few minutes ago, this research is to better understand why people go to see drug sellers and private providers when their children have respiratory problems, how private providers diagnose and treat these problems, and how caregivers then apply the advice they receive. The information we learn will help find ways to better help sick children in Uttar Pradesh. We would like to talk with you today about your experiences with children with respiratory illnesses.

**General background**

Before we get started, I would like to talk about yourself.

- Could you tell me a bit about yourself and about your experience as a drug seller?
    a. *Socio-demographic characteristics characteristics have been covered*
    b. How long have you been working in this position?
    c. What services do you currently provide?
- How does the community learn about you?
- How often do people come to see you in relation to illness among children under the age of five?
    a. When people with sick children come to see you, how common is it for them to bring their child along?
    b. What are some of the other places, if any, that they typically go to for child illness before they come to you?

**Diagnosis**

Thank you. Now I would like to talk about your experience with children with respiratory illnesses.

- What different respiratory illnesses do you see among children under the age of five in your clientele? At what time of the year do you see the most cases?
- In your experience, how often do caregivers of children with respiratory illnesses come directly to you without going to see a healthcare provider first?
  *If caregivers sometimes come directly to drug seller:*
    a. What do you think are some of the reasons why they come to you directly?
    b. What kind of help do they ask you for?
        i. *Clarify diagnosis vs. prescribe treatment vs. just ask for specific drugs*
    c. What do you do to better understand what illness the child has?
        *i. Probe for specific questions asked and for any job aides or diagnostic tools they may use*

**Understanding and treatment of pneumonia**

- What do you call pneumonia in this area?
    a. What are the ways to tell when a child has pneumonia and not some other illness?
    b. Are there different types of pneumonia?
        i. *If yes:* What are the differences between them?
- To what extent do you feel that children who are sick with pneumonia may be able to make a full recovery without taking any drugs? Explain.
- In your experience, what is the most effective treatment for a child with pneumonia?
    a. What is the most effective drug or combination of drugs? *Probe for drugs vs. no drugs, alone or in combination, duration, dose. Probe for name of drug and salt. If possible, ask to see drug at the end of the interview and note name, composition, and dose. If no drugs, probe about other actions.*

- Have there been times when caregivers came to you with a prescription for a child with pneumonia and you ended up selling them a substitute?
  
  _If yes:_
  a. What are some of the reasons why you sold them a substitute? *Cost, availability etc.*
  b. What are some of the substitutes that you have sold? *Probe for name of drug and salt, dosage, duration (kit dose).*
- Have there been times when caregivers asked for your advice on how to treat a child who was sick with pneumonia? When was the last time this happened?
  a. What were the circumstances?
  b. What did you tell them to do?
- Have you ever seen a child with pneumonia that was so severe that you thought that this child needed to see a doctor? When was the last time this happened?
  a. What were the circumstances? Why are the reasons why you felt this child needed help?
  b. Where did you tell the caregiver to go? Why?
     i. *Establish point of referral, including type (public vs. private and level – health facility, hospital; or private provider type – allopathic, ISMP, or other – ASHA, anganwadi)*

**Drug dispensing**

- Where do you get the drugs that you sell that can treat pneumonia?
  a. *Establish whether single vs. multiple suppliers, and supplier type (wholesaler, sales representatives for drug companies)*
  b. What do you like about your current supplier(s)? What do you not like?
- How do you decide what drugs to buy? What are the different factors that you consider when making this decision?
- Have there been times when you wanted to dispense some drugs to someone caring for a child with pneumonia but you were out of stock? When was the last time this happened?
  a. What were the circumstances?
  b. What did you do?

**Amoxicillin**

- Have you ever sold amoxicillin for any child illness?
  
  _If yes_:
  a. What illnesses did you sell it for?
  b. What delivery form(s) of amoxicillin do you sell the most for children (syrup, dry powder for reconstitution with water to form a suspension, dispersible tablet)? Why do you think that this form is more popular than others for children?
     i. *Cost, convenience, acceptability*
- In general, what do you think is the most suitable delivery form for drugs for children (e.g. syrup, drops, powder, tablets, dispersible tablets)?
  a. What do you think are the advantages of dispersible tablets over other delivery forms?
  b. What are the disadvantages?

**Final words**

- Where do you get your information about child illnesses?
  a. What would be the best way for you to receive updates on child illnesses given your current busy schedule?
- Overall, what do you find most challenging about knowing the best way to help children with respiratory illnesses?
  a. What additional information or tools do you think you need to better help these children?
- Do you have any final questions or recommendations to make to improve the management of child respiratory illnesses in this area?
